# Mature Human Milk Macronutrient Composition and Apparent Milk TSH Concentration in Women with Treated Hypothyroidism Attending Lactation Counseling: A Cross-Sectional Study

**DOI:** 10.3390/metabo16090656

**Published:** 2026-09-08

**Authors:** Urszula Bernatowicz-Łojko, Elena Sinkiewicz-Darol, Maria Wilińska, Barbara Baranowska, Liliana Pięta, Renata Gadzała-Kopciuch

**Affiliations:** 1 Department of Midwifery, Centre of Postgraduate Medical Education, 01-004 Warsaw, Poland; barbara.baranowska@cmkp.edu.pl (B.B.); liliana.pieta@cmkp.edu.pl (L.P.); 2 Ludwik Rydygier’ Provincial Polyclinical Hospital, Human Milk Bank, 87-100 Toruń, Poland; elena.sinkiewicz-darol@ukw.edu.pl; 3 Department of Physiology and Toxicology, Faculty of Biological Sciences, Kazimierz Wielki University, 85-064 Bydgoszcz, Poland; 4 Department of Obstetrics and Perinatology, National Medical Institute of the Ministry of the Interior and Administration, 02-507 Warsaw, Poland; wilinska.maria@gmail.com; 5 Department of Environmental Chemistry and Bioanalytics, Faculty of Chemistry, Nicolaus Copernicus University in Toruń, 87-100 Toruń, Poland; rgadz@umk.pl

**Keywords:** human milk, treated hypothyroidism, Hashimoto’s disease, lactation, macronutrients, hyperlactation, pre-pregnancy BMI, apparent milk TSH, human milk bank

## Abstract

**Highlights:**

**What are the main findings?**

- In this selected cohort of women attending lactation counseling/human milk-bank consultation, a documented diagnosis/history of treated or clinically managed hypothyroidism was not associated with statistically significant differences in mature human milk macronutrient composition compared with controls without known thyroid disorders.
- Exploratory analyses did not identify statistically significant associations between apparent milk TSH concentration and pre-pregnancy BMI, hyperlactation, or macronutrient composition; these analyses were limited by sample size, sparse subgroups, and lack of milk-matrix assay validation.

**What are the implications of the main findings?**

- The findings may support cautious breastfeeding counseling in this selected population, but they should not be interpreted as evidence of equivalence, confirmed euthyroidism during milk sampling, or absence of smaller biological effects.
- Apparent milk TSH measurements should be interpreted cautiously because no study blood sampling was performed at milk collection, contemporaneous maternal serum TSH/FT4 values and levothyroxine dose/timing data were not available, and formal validation of the assay in human milk was not performed.

**Abstract:**

Background/Objectives: Hypothyroidism may influence lactation physiology, but evidence regarding its association with mature human milk composition remains limited. This cross-sectional study evaluated macronutrient composition and apparent thyroid-stimulating hormone (TSH) concentration in 24 h composite mature milk samples from women with a documented history/diagnosis of treated or clinically managed hypothyroidism attending lactation counseling and human milk-bank consultation, compared with controls without known thyroid disorders. Methods: Sixty-six lactating women attending the Human Milk Bank in Toruń, Poland, were enrolled (31 with hypothyroidism, including six with Hashimoto’s disease; 35 controls). Thyroid characterization was based on diagnoses and laboratory results documented during routine preconception or early-pregnancy/perinatal care; the study did not include contemporaneous maternal serum TSH/FT4 testing at milk collection in either group. Composite 24 h milk samples were analyzed using the MIRIS Human Milk Analyzer for fat, crude protein, true protein, carbohydrates, total solids, and energy. Milk TSH was measured using a third-generation ELFA assay. Because this serum/plasma assay was not formally validated for the human milk matrix, values are reported as exploratory apparent milk TSH concentrations. Results: No statistically significant between-group differences were observed in macronutrient composition in the analyses performed. For example, the HG-CG mean difference for energy content was −3.47 kcal/100 mL (95% CI: −8.45 to 1.52; *p* = 0.169; q = 0.814), and all confidence intervals for milk-composition outcomes included zero. Median apparent milk TSH concentration was 0.0180 [IQR 0.0130–0.0208] µIU/mL in controls and 0.0155 [IQR 0.0093–0.0210] µIU/mL in the hypothyroidism group (Mann–Whitney U = 459.0, *p* = 0.286). Hyperlactation was frequent in this selected cohort and was more common in controls (27/35; 77.1%) than in the hypothyroidism group (16/31; 51.6%). Exploratory analyses of pre-pregnancy BMI and Hashimoto’s disease were limited by small subgroup sizes. Conclusions: In this small selected cohort, a documented diagnosis/history of treated or clinically managed hypothyroidism was not associated with statistically significant differences in mature milk macronutrient composition or apparent milk TSH concentration. These findings are exploratory and should not be interpreted as evidence of equivalence, confirmed euthyroidism at sampling, or exclusion of smaller effects.

## 1. Introduction

Human milk is a dynamic biological fluid whose composition reflects maternal physiology, infant needs, lactation stage, and sampling methodology. Although macronutrient content tends to be more stable during established lactation than in colostrum or transitional milk, variation may still occur in relation to metabolic status, endocrine function, inflammation, and milk expression practices [1,2]. Thyroid hormones are key endocrine regulators of metabolism, energy expenditure, mammary gland development, and lactation physiology; adequate thyroid function is also important for infant growth and neurodevelopment [3].

Hypothyroidism is among the most common endocrine disorders in women of reproductive age and may persist into the postpartum period. Untreated or insufficiently treated hypothyroidism has been associated with delayed lactogenesis, reduced milk volume, and impaired milk ejection [4,5]. Potential mechanisms include altered prolactin signaling, disrupted lipid, protein, and carbohydrate metabolism, and reduced mammary gland responsiveness [6]. However, these mechanisms do not necessarily imply altered macronutrient composition in mature milk when hypothyroidism has been diagnosed and treated in routine care.

Previous studies examining thyroid dysfunction and human milk have often focused on colostrum or early lactation, used single milk samples, or did not fully account for maternal treatment status, iodine exposure, body mass index, hyperlactation, or pregnancy-related comorbidities [7,8,9,10]. In routine clinical practice, many women with hypothyroidism receive levothyroxine therapy before or during pregnancy and continue medical supervision during lactation [11]. Therefore, the specific question addressed here is not whether current biochemical hypothyroidism changes milk composition, but whether a documented diagnosis/history of treated or clinically managed hypothyroidism is associated with mature milk macronutrient composition in a selected lactation-counseling setting.

Thyroid-stimulating hormone (TSH), thyroxine, and other thyroid-related hormones have been detected in human milk [12,13,14,15,16]. A recent study showed that TSH and thyroxine are measurable in own mother’s milk and donor milk during the first months of lactation, but three clinically distinct questions remain unresolved: whether TSH can be reliably detected in human milk with assays validated for this matrix, whether milk TSH reflects contemporaneous maternal circulating thyroid status, and whether milk TSH has physiological effects in the breastfed infant [16]. Because the assay used in the present study was not formally validated for human milk, we refer to these measurements as apparent milk TSH concentrations and do not treat them as a substitute for maternal serum TSH or FT4 assessment.

This study aimed to compare the macronutrient composition and apparent milk TSH concentration of mature 24 h composite human milk samples from women with a documented diagnosis/history of treated or clinically managed hypothyroidism, including a small exploratory subgroup with Hashimoto’s disease, with samples from women without known thyroid disorders. Secondary analyses explored pre-pregnancy BMI and clinically defined hyperlactation because both may be linked to endocrine–metabolic status, lactation physiology, and referral patterns in lactation-counseling populations. By focusing on established lactation and 24 h composite sampling, the study addresses a gap left by earlier work based mainly on early lactation samples or less standardized collection procedures, while requiring cautious interpretation because the cohort was clinically selected rather than population-based.

## 2. Materials and Methods

### 2.1. Study Design and Participants

This observational, cross-sectional study included 66 lactating women who sought lactation counseling and/or human milk-bank consultation at the Human Milk Bank of the Ludwik Rydygier Provincial Polyclinical Hospital in Toruń, Poland, between March 2020 and November 2021. Participants were recruited during routine consultations and provided written informed consent before enrollment. All participants were in established lactation at the time of sampling (≥4 weeks postpartum). Therefore, no early-vs-established lactation subgroup analysis was performed. Participant flow was simple within the analytic dataset: 66 women were enrolled, no enrolled participant was excluded from the milk-composition or apparent milk TSH analyses, and all 66 had complete outcome data. The number of women approached but not enrolled during routine counseling was not systematically recorded.

Participants were recruited from a clinical lactation counseling and human milk-bank setting rather than from a population-based cohort. Thus, the sample represents women seeking lactation support and/or milk-bank consultation. The detailed indication for consultation was not collected as a mutually exclusive analyzable variable for every participant; however, the observed clinical context included breastfeeding difficulties such as excessive milk production or forceful flow, infant choking or regurgitation during feeding, milk-transfer or breastfeeding-management concerns, and questions about milk quality or possible donation. This selected recruitment setting was considered when interpreting the high prevalence of hyperlactation and the external validity of the findings.

Two groups were defined: the hypothyroidism group (HG; *n* = 31), comprising women with a documented history/diagnosis of hypothyroidism, including Hashimoto’s disease (*n* = 6), and the control group (CG; *n* = 35), comprising women without known thyroid disorders. In this manuscript, “treated” or “clinically managed” hypothyroidism means a documented diagnosis established before conception or during early pregnancy/perinatal care, with routine medical follow-up and levothyroxine therapy when clinically indicated; it does not mean that biochemical euthyroidism was confirmed at the time of milk sampling. Thyroid disorders in the HG were diagnosed on the basis of clinical evaluation, medical documentation, and laboratory testing performed as part of routine preconception or early-pregnancy/perinatal care (including TSH, FT4, and thyroid autoantibodies when clinically indicated) [17,18], following endocrinology guidelines [19,20,21]. No blood samples for thyroid hormone assessment were collected specifically for this study at the time of milk sampling. The CG was classified according to the absence of self-reported or documented thyroid disease and absence of thyroid-specific treatment; contemporaneous serum TSH/FT4 or antibody testing was not performed in controls, so undiagnosed thyroid dysfunction cannot be excluded.

None of the participants received individual therapeutic doses of iodine. During pregnancy and lactation, participants reported use of preparations containing prophylactic iodine doses, typically 150–200 µg/day, as recommended in Poland; exact product names, adherence, urinary iodine concentration, and breast-milk iodine concentration were not recorded. In addition, Poland has maintained a national iodine prophylaxis model based on obligatory iodization of household salt since 1997; the iodine content of salt has been described as 30 ± 10 mg potassium iodide/kg NaCl or, since 2002, 39 ± 13 mg potassium iodate/kg NaCl [22]. Inclusion criteria were age ≥ 18 years, singleton pregnancy, term or near-term birth (≥37 weeks), established lactation, and ability to provide a complete 24 h milk sample. Exclusion criteria were postpartum thyroiditis, untreated thyroid dysfunction, other endocrine disorders (e.g., type 1 diabetes, PCOS), medications known to affect lactation (dopaminergic agents, corticosteroids), active mastitis, smoking or substance use, and chronic diseases affecting metabolism (renal, hepatic, or autoimmune disease other than Hashimoto’s disease).

Sociodemographic and clinical data were collected using a structured 43-item questionnaire covering maternal characteristics, pregnancy course, mode of birth, infant feeding, chronic diseases, medication use, and lifestyle factors. Pre-pregnancy BMI was calculated using pre-pregnancy weight and height; BMI ≥ 25 kg/m^2^ was classified as overweight/obesity according to the WHO adult BMI classification [23].

Hyperlactation (HL) was defined clinically because the literature does not provide a single universally accepted diagnostic threshold. In this study, HL should be understood as a composite clinical syndrome of perceived oversupply and/or forceful milk ejection with infant feeding symptoms, rather than as an objectively measured milk-volume phenotype. HL was assessed by a lactation consultant and recorded when persistent symptoms suggested excessive milk production or excessive milk flow. Criteria included intense milk flow during latch or feeding, milk leaking from the infant’s mouth during breastfeeding, audible rapid swallowing suggestive of air swallowing while attempting to slow milk flow, choking or coughing during feeding, the infant being “flooded” with milk during breastfeeding, excessive infant weight gain relative to age norms, and/or marked regurgitation or burping after feeds after exclusion of other causes, such as sonographic features of pyloric stenosis. This definition is consistent with the clinical framing of the Academy of Breastfeeding Medicine, which recognizes hyperlactation as a condition that may be self-induced, iatrogenic, or idiopathic [24].

### 2.2. Milk Sampling

Milk collection followed standardized written instructions. Each participant collected milk over a 24 h period in four intervals: 06:00–12:00, 12:00–18:00, 18:00–24:00, and 24:00–06:00. Completion was checked by confirming that milk from all four intervals had been provided; however, exact time stamps, total expressed volume, and independent monitoring of home collection adherence were not recorded.

During each interval, mothers expressed 10–15 mL of milk before and after breastfeeding. For exclusively pumping mothers, 15 mL aliquots were taken from each expressed portion. Samples were stored at 4–8 °C and transported to the laboratory in a cooled container. The maximum storage time before laboratory processing was 12 h.

For each participant, all interval samples were pooled into a single composite sample representing the 24 h period. Macronutrient analysis was performed on the fresh pooled composite sample. An aliquot of the same pooled sample was stored at −80 °C and later used for apparent milk TSH measurement. Repeated freeze–thaw cycles were not part of the protocol, but the exact number of freeze–thaw events was not recorded as a study variable.

### 2.3. Determination of Macronutrient Composition

Macronutrient content—fat, crude protein, true protein, carbohydrates, total solids, and energy—was analyzed using the MIRIS Human Milk Analyzer (Miris AB, Uppsala, Sweden), based on mid-infrared transmission spectroscopy [25]. The analyzer reports crude protein as a nitrogen-derived protein-equivalent estimate that includes both protein and non-protein nitrogen fractions, whereas true protein is corrected for non-protein nitrogen and may be more directly interpretable nutritionally. Energy content was reported by the analyzer using its macronutrient-based calculation algorithm. This method is widely used in clinical and milk-bank settings for rapid human milk macronutrient assessment, although validation studies indicate that accuracy varies by analyte and that protein measurements require cautious interpretation [25,26].

Before analysis, samples were heated to 40 °C and homogenized using a MIRIS Sonicator (1.5 s/mL). Each sample was analyzed in triplicate, and the mean value was used for statistical analysis. Daily zero-calibration was performed using manufacturer-provided standards.

### 2.4. Measurement of TSH Concentration in Human Milk

Apparent milk TSH concentrations were determined using a third-generation enzyme immunofluorescence assay (ELFA) on the VIDAS analyzer (bioMérieux, Marcy-l’Étoile, France), following the manufacturer’s analytical protocol [27]. Milk samples were centrifuged at 2000 rpm for 5 min, and 200 µL of supernatant was used for analysis. Each measurement was performed in duplicate. The assay is intended and manufacturer-validated for human serum or plasma rather than human milk; therefore, milk TSH values in this study should be interpreted as exploratory matrix measurements. No formal recovery, linearity, dilution, protein-binding, or fat-content interference validation in the milk matrix was performed. The manufacturer-reported measuring range for the serum/plasma TSH3 assay is very low, but the limit of detection, limit of quantification, and recovery characteristics cannot be assumed to apply to human milk. Values returned as 0.0000 µIU/mL by the analyzer/software were retained as recorded in non-parametric descriptive analyses.

### 2.5. Statistical Analysis

All statistical analyses were performed using IBM SPSS Statistics 26.0 (IBM Corp., Armonk, NY, USA). The distribution of continuous variables was assessed using the Shapiro–Wilk test and visual inspection of histograms and Q–Q plots. Approximately normally distributed variables were analyzed using parametric tests (*t*-test or ANOVA), whereas non-normally distributed variables were analyzed using non-parametric methods (Mann–Whitney U or Kruskal–Wallis tests).

The six milk-composition outcomes constituted the primary outcome family. The between-group comparison of apparent milk TSH concentration was treated as a single secondary outcome. Pre-pregnancy BMI, hyperlactation, Hashimoto’s disease, and correlation analyses were considered exploratory and hypothesis-generating because of the modest sample size and sparse subgroup counts.

Baseline continuous characteristics were compared using Welch’s two-sample *t* tests and are reported together with the HG−CG mean difference and its 95% confidence interval. Categorical characteristics were compared using Pearson’s chi-square test, whereas Fisher’s exact test was used for GDM and PIH because of small expected cell counts. Effect estimates for categorical variables were expressed as odds ratios comparing the HG with the CG, with corresponding 95% confidence intervals. Baseline comparisons were descriptive and were not included in the false discovery rate correction. Lactation stage was not modeled as an early-vs-established variable because all participants were already in established lactation. Between-group comparisons of the six milk-composition outcomes were performed using Welch’s two-sample *t* tests, which do not assume equal variances. For each outcome, the mean difference between groups (HG − CG) was reported together with its two-sided 95% confidence interval calculated using the Welch–Satterthwaite degrees of freedom, the exact *p* value, and Hedges’ g. Hedges’ g was calculated using the pooled within-group standard deviation with a small-sample correction; negative values indicate lower mean values in the HG than in the CG. To control the false discovery rate across the family of six milk-composition outcomes, *p* values were adjusted using the Benjamini–Hochberg procedure and are reported as q values. Differences in the prevalence of hyperlactation, GDM, PIH, and BMI category were assessed using Chi-square tests or Fisher exact tests when expected cell counts were small. Subgroup analyses of BMI, hyperlactation, and Hashimoto’s disease were exploratory because of the modest sample size and sparse subgroup counts.

Separate two-way ANOVA models were fitted for each milk-composition outcome, with study group (HG vs. CG), pre-pregnancy BMI category (<25 vs. ≥25 kg/m^2^), and the group × pre-pregnancy BMI interaction included as model terms. Homogeneity of variance was assessed using Levene’s test. Effect sizes were expressed as partial eta squared (η^2^p). To control the false discovery rate across the six pre-pregnancy BMI main-effect tests, the corresponding *p* values were adjusted using the Benjamini–Hochberg procedure. Interaction and subgroup findings were interpreted cautiously because of the modest sample size. Because HL was highly prevalent and unevenly distributed by recruitment group, and because subgroup cells were small, formal multivariable adjustment for HL was not performed; instead, HL findings were treated as descriptive and exploratory.

Because apparent milk TSH concentrations were not normally distributed, they were summarized using the median, interquartile range (IQR), and minimum–maximum range. Between-group comparisons were performed using the Mann–Whitney U test, and differences across four subgroups defined by study group and pre-pregnancy BMI category or hyperlactation status were assessed using the Kruskal–Wallis H test. For the main comparison between the HG and CG, the between-group location shift was estimated using the Hodges–Lehmann estimator, defined as the median of all pairwise differences (HG − CG). Its two-sided 95% confidence interval was calculated using the normal approximation to the Wilcoxon rank-sum statistic with correction for ties and continuity. Effect sizes for Mann–Whitney comparisons based on the asymptotic test were expressed as r, calculated as |Z|/√N. For comparisons involving small or unbalanced subgroups, including the Hashimoto’s disease subgroup, the exact Mann–Whitney U test was used where appropriate, and the effect size was expressed as the rank-biserial correlation. Effect sizes for Kruskal–Wallis tests were expressed as eta squared based on the H statistic (η^2^H). Associations between apparent milk TSH concentrations and macronutrient content were evaluated using Spearman’s rank-order correlation. To control the false discovery rate across the 12 Spearman correlation tests (six milk-composition outcomes evaluated separately in the two study groups), *p* values were adjusted using the Benjamini–Hochberg procedure and are reported as q values.

No a priori sample-size calculation was performed. The study sample comprised all eligible consenting participants enrolled during the study period. A sensitivity analysis indicated that, with 31 participants in the hypothyroidism group and 35 participants in the control group, a two-sided independent two-sample comparison at α = 0.05 had 80% power to detect a standardized between-group difference of approximately Cohen’s d = 0.70. Therefore, the study may not have detected small or moderate between-group differences and was not designed to establish equivalence or non-inferiority.

All statistical tests were two-sided, and statistical significance was set at *p* < 0.05. Effect sizes were reported as Hedges’ g for between-group comparisons of milk composition, partial eta squared (η^2^p) for ANOVA effects, eta squared based on the Kruskal–Wallis statistic (η^2^H), and r or rank-biserial correlation for Mann–Whitney comparisons, as appropriate. Adjusted *p* values obtained using the Benjamini–Hochberg procedure are reported as q values. No formal equivalence or non-inferiority analysis with pre-defined clinical margins was performed; therefore, non-significant findings should not be interpreted as evidence of equivalence or absence of an effect. Secondary and subgroup analyses were considered exploratory.

## 3. Results

### 3.1. Participant Characteristics

A total of 66 lactating women were included 31 in the HG with a documented history/diagnosis of hypothyroidism (including Hashimoto’s disease) and 35 in the CG without known thyroid disorders. All enrolled women contributed complete milk macronutrient and apparent milk TSH data. The groups did not differ significantly in maternal age, gestational age at birth, mode of birth, parity, or stage of lactation at the time of sampling (Table 1). Information on eligible women who were not enrolled during routine counseling was not recorded systematically, which limits participant-flow reporting.

Hypothyroidism was diagnosed before conception or during the first trimester in most women in the HG. Levothyroxine therapy was used by 25/31 women (81%) during pregnancy and by 19/31 (61%) during lactation; all six women with Hashimoto’s disease received treatment throughout the study period [19,20,21]. Exact dose ranges, treatment categories, most recent thyroid-function test timing, and medication timing relative to milk collection were not available in the research dataset; therefore, the HG should be interpreted as women with a history/diagnosis of clinically managed hypothyroidism, not as women with confirmed biochemical hypothyroidism or confirmed euthyroidism at milk sampling.

GDM was more frequent in the HG (7/31; 22.6%) than in the CG (4/35; 11.4%), but this difference was not statistically significant (Fisher’s exact test, *p* = 0.324). PIH occurred in 3/31 women (9.7%) in the HG and 5/35 women (14.3%) in the CG (Fisher’s exact test, *p* = 0.713). Overweight/obesity (BMI ≥ 25 kg/m^2^) affected more than 30% of women in both groups before pregnancy and after birth.

Hyperlactation was significantly more common in the control group (27/35; 77.1%) than in the hypothyroidism group (16/31; 51.6%) (χ^2^ = 4.719, *p* = 0.030). These are within-group prevalences; the previously reported 62.8% and 37.2% values referred to the distribution of all hyperlactation cases across groups and have been removed to avoid misunderstanding. Because the cohort was recruited through lactation counseling and milk-bank consultation, this high prevalence should be interpreted as a feature of the selected clinical sample rather than as a population estimate.

### 3.2. TSH Concentrations in Human Milk

Because apparent milk TSH concentrations were non-normally distributed, the results are presented as medians, interquartile ranges, and minimum–maximum ranges. The median apparent milk TSH concentration was 0.0180 [0.0130–0.0208] µIU/mL in the control group (CG; *n* = 35; range: 0.0025–0.0690 µIU/mL) and 0.0155 [0.0093–0.0210] µIU/mL in the hypothyroidism group (HG; *n* = 31; range: 0.0000–0.1080 µIU/mL). The Hodges–Lehmann estimate of the between-group location shift (HG − CG) was −0.0020 µIU/mL (95% CI: −0.0070 to 0.0020 µIU/mL). The Mann–Whitney U test showed no statistically significant difference between the groups (U = 459.0, Z = −1.07, *p* = 0.286; r = 0.131). Because the assay was not validated for milk, these numerical values should be regarded as apparent matrix measurements rather than established physiological milk TSH concentrations.

Within the HG, an exploratory comparison was conducted between women with Hashimoto’s disease (*n* = 6) and women with hypothyroidism without Hashimoto’s disease (*n* = 25). The median apparent milk TSH concentration was 0.0100 [0.0091–0.0124] µIU/mL in women with Hashimoto’s disease (range: 0.0000–0.0235 µIU/mL) and 0.0170 [0.0100–0.0210] µIU/mL in women with hypothyroidism without Hashimoto’s disease (range: 0.0000–0.1080 µIU/mL). An exploratory comparison using the exact Mann–Whitney U test showed no statistically significant difference in apparent milk TSH concentrations between women with Hashimoto’s disease and women with hypothyroidism without Hashimoto’s disease (U = 49.0, exact *p* = 0.208; rank-biserial correlation = −0.347). Given the small number of participants with Hashimoto’s disease, this subgroup comparison should be regarded as exploratory and hypothesis-generating.

### 3.3. Macronutrient Composition of Human Milk

Macronutrient content of mature milk did not differ significantly between groups in the analyses performed (Table 2).

Welch’s two-sample *t* tests showed no statistically significant between-group differences in any of the six milk-composition outcomes. The smallest *p* value was observed for energy content (*p* = 0.169), and q values after Benjamini–Hochberg correction ranged from 0.814 to 0.961. The largest absolute standardized effect was also observed for energy content (Hedges’ g = −0.345), but the 95% confidence interval for the mean difference included zero. These results should not be interpreted as evidence of equivalence or absence of smaller effects.

### 3.4. Associations Between TSH and Milk Composition

Spearman’s rank-order correlation identified a nominal positive association between apparent milk TSH concentration and carbohydrate content in the CG (ρ = 0.363, *p* = 0.032), but this association did not remain statistically significant after Benjamini–Hochberg correction across the 12 correlation tests (q = 0.387). No other correlation was nominally significant (all *p* values were ≥0.122), and none remained statistically significant after false discovery rate correction (q values ≥ 0.387). Given the absence of concurrent maternal serum hormone data and limited milk-matrix validation of the TSH assay, these findings should not be interpreted as evidence that milk TSH reflects maternal thyroid status.

### 3.5. Associations of Pre-Pregnancy BMI with Human Milk TSH and Milk Composition

Pre-pregnancy BMI was categorized as <25 or ≥25 kg/m^2^, combining overweight and obesity into a single category because of the modest sample size. The resulting subgroups comprised CG/pre-pregnancy BMI < 25 (*n* = 24), CG/pre-pregnancy BMI ≥ 25 (*n* = 11), HG/pre-pregnancy BMI < 25 (*n* = 20), and HG/pre-pregnancy BMI ≥ 25 (*n* = 11). Apparent milk TSH concentrations did not differ significantly among these four subgroups (Kruskal–Wallis H(3) = 2.73, *p* = 0.436; η^2^H = 0.00).

Separate two-way ANOVAs were conducted for fat, crude protein, carbohydrates, total solids, true protein, and energy content, with study group (HG vs. CG) and pre-pregnancy BMI category as factors. No group × pre-pregnancy BMI interaction was observed for fat (F(1,62) = 0.38, *p* = 0.539, η^2^p = 0.006), crude protein (F(1,62) = 0.04, *p* = 0.838, η^2^p = 0.001), carbohydrates (F(1,62) = 0.45, *p* = 0.503, η^2^p = 0.007), total solids (F(1,62) = 0.46, *p* = 0.502, η^2^p = 0.007), true protein (F(1,62) = 0.06, *p* = 0.803, η^2^p = 0.001), or energy content (F(1,62) = 0.09, *p* = 0.761, η^2^p = 0.002). No significant main effects of study group were identified in these exploratory models.

Before correction for multiple comparisons, a main effect of BMI category was observed for energy content (F(1,62) = 4.91, *p* = 0.030, η^2^_*p* = 0.073). However, this association did not remain significant after Benjamini–Hochberg correction across the six BMI main-effect tests (q = 0.181). The main effects of BMI were also non-significant for fat (F(1,62) = 3.66, *p* = 0.060), crude protein (F(1,62) = 0.10, *p* = 0.753), carbohydrates (F(1,62) = 0.34, *p* = 0.562), total solids (F(1,62) = 2.32, *p* = 0.133), and true protein (F(1,62) = 0.21, *p* = 0.650). Thus, no milk-composition outcome showed a statistically significant association with pre-pregnancy BMI after correction for multiple testing.

Hyperlactation occurred in 14 of 22 women with pre-pregnancy BMI ≥ 25 kg/m^2^ (63.6%) and in 29 of 44 women with pre-pregnancy BMI < 25 kg/m^2^ (65.9%). This difference was not statistically significant (Fisher’s exact test, *p* = 1.000).

### 3.6. Association of Hyperlactation with Apparent Milk TSH Concentrations

Apparent milk TSH concentrations were compared across four subgroups defined by study group and hyperlactation status: CG without hyperlactation (*n* = 8), CG with hyperlactation (*n* = 27), HG without hyperlactation (*n* = 15), and HG with hyperlactation (*n* = 16). The corresponding median apparent milk TSH concentrations [IQR] were 0.0133 [0.0096–0.0261], 0.0185 [0.0143–0.0203], 0.0105 [0.0045–0.0180], and 0.0183 [0.0124–0.0215] µIU/mL, respectively. The Kruskal–Wallis test showed no statistically significant differences among the four subgroups (H(3) = 4.69, *p* = 0.196; η^2^H = 0.027).

In the overall sample, the median apparent milk TSH concentration was 0.0185 [0.0135–0.0210] µIU/mL among women with hyperlactation and 0.0125 [0.0068–0.0220] µIU/mL among women without hyperlactation. This difference did not reach statistical significance (Mann–Whitney U = 637.0, *p* = 0.056; rank-biserial correlation = 0.288). Exploratory analyses conducted separately within the study groups also showed no significant association between hyperlactation and apparent milk TSH concentration (CG: U = 123.5, *p* = 0.555; HG: U = 162.0, *p* = 0.101). Therefore, the available data did not provide evidence of an association between hyperlactation and apparent milk TSH concentration, but the imbalance in hyperlactation between groups remains an important interpretive limitation (Table 3).

## 4. Discussion

### 4.1. Principal Findings

In this selected cross-sectional cohort of women attending lactation counseling and human milk-bank consultation, a documented diagnosis/history of treated or clinically managed hypothyroidism was not associated with statistically significant differences in the measured mature milk macronutrients. Confidence intervals for all between-group milk-composition differences included zero; the largest standardized difference was observed for energy content, but the estimate was imprecise and should not be interpreted as evidence of equivalence or as excluding smaller effects.

### 4.2. Comparison with Previous Studies and Metabolic Framing

Previous studies of thyroid dysfunction and human milk have used heterogeneous designs, including early lactation samples, different thyroid-disease definitions, and variable control of treatment status and iodine exposure [7,8,9,10]. The present study contributes a focused clinical milk-composition dataset based on mature 24 h composite samples. Although it is not a metabolomics study, it is relevant to the scope of endocrine–metabolic lactation research because hypothyroidism may influence lipid, protein, carbohydrate, and energy metabolism through systemic and mammary-gland pathways.

### 4.3. Interpretation of Apparent Milk TSH Findings

Apparent milk TSH concentrations were low and were not significantly different between women with a documented history/diagnosis of treated or clinically managed hypothyroidism and controls without known thyroid disease. These findings should be interpreted within three separate questions: first, whether TSH is detectable in human milk; second, whether milk TSH reflects maternal circulating thyroid status; and third, whether milk TSH has physiological relevance for the infant. The current study contributes only to the first question in an exploratory way and cannot answer the second or third questions because no contemporaneous maternal serum TSH/FT4 testing was performed and the assay was not formally validated for the human milk matrix. Matrix interference, protein binding, fat content, dilution/recovery effects, analytical sensitivity at very low concentrations, and interference from milk components may all affect the apparent concentrations obtained with a serum/plasma assay. Therefore, the numerical values reported here should not be treated as quantitatively established physiological milk TSH concentrations.

### 4.4. Hyperlactation, Pre-Pregnancy BMI, and Exploratory Variables

Hyperlactation was frequent and significantly more common in the control group than in the hypothyroidism group. This finding is best interpreted as a characteristic of the selected counseling/milk-bank cohort rather than as evidence for or against a biological effect of hypothyroidism. The operational definition of hyperlactation used here reflected a composite clinical syndrome of perceived oversupply and/or forceful milk ejection with infant feeding symptoms. These phenomena are clinically related but not identical to objectively measured milk production. Because reasons for lactation consultation, infant feeding practices, and milk-donation interests may themselves be related to milk volume and milk composition, hyperlactation may have introduced selection bias or residual confounding. Pre-pregnancy BMI analyses were also exploratory and did not remain significant after false discovery rate correction.

### 4.5. Strengths and Limitations

The main strengths of this study include use of mature milk, standardized 24 h composite sampling, triplicate macronutrient analysis, and transparent reporting of exploratory analyses. Several limitations are important. First, the sample was small and highly selected, and women attending lactation counseling or a human milk bank may differ systematically from the broader breastfeeding population. Second, no serum TSH, FT4, or antibody measurements were obtained at milk sampling; therefore, the study compares women with a documented diagnosis/history of hypothyroidism rather than women with confirmed biochemical thyroid status at lactation. Third, the control group was defined by absence of known thyroid disease and thyroid-specific treatment, but occult thyroid dysfunction cannot be excluded. Fourth, levothyroxine dose, medication timing relative to milk collection, exact timing of the most recent thyroid-function test, diet, iodine status, urinary iodine, and breast-milk iodine concentration were not available. Fifth, collection adherence was based on receipt of complete interval samples rather than independently verified time-stamped collection. Sixth, the TSH assay was not validated in human milk, and milk-matrix effects could influence the apparent values. Finally, the statistical analyses involving Hashimoto’s disease, pre-pregnancy BMI, hyperlactation, and correlations were exploratory and may be underpowered.

### 4.6. Clinical Implications and Future Research

These results may provide limited reassurance that large differences in the measured mature milk macronutrients were not detected in this selected, predominantly treated cohort. However, the findings should not be generalized to untreated or suboptimally treated hypothyroidism, to women with confirmed biochemical hypothyroidism during lactation, or to the general breastfeeding population. Future studies should include larger and more diverse cohorts, prospective recruitment, contemporaneous maternal serum TSH/FT4 and thyroid antibody measurements, detailed levothyroxine dose and timing data, iodine-status assessment, dietary assessment, formal milk-matrix validation of thyroid hormone assays, and infant outcome data.

## 5. Conclusions

In this small selected cohort of women attending lactation counseling and human milk-bank consultation, a documented diagnosis/history of treated or clinically managed hypothyroidism, including an exploratory subgroup with Hashimoto’s disease, was not associated with statistically significant differences in the measured mature milk macronutrient composition or apparent milk TSH concentration. The study did not confirm euthyroidism at milk sampling, was not powered to detect small or moderate differences, and was not designed to establish equivalence.

Future research should use larger population-based and clinical cohorts, contemporaneous thyroid-function testing, detailed treatment and iodine-status data, validated milk-matrix assays for thyroid-related biomarkers, and longitudinal infant outcomes to clarify whether maternal thyroid function during lactation has clinically meaningful associations with human milk composition.

## Figures and Tables

**Table 1:** Demographic and clinical characteristics of the study groups.

Study Parameter	Control Group CG (*n* = 35)	Hypothyroidism Group HG (*n* = 31)	Effect Estimate (95% CI)	Test; *p* Value
Maternal age (years), mean ± SD	31.1 ± 4.0 (*n* = 35)	31.5 ± 3.5 (*n* = 31)	MD 0.41 (−1.42 to 2.23)	Welch t; 0.658
Lactation period (days), mean ± SD	62.4 ± 34.8 (*n* = 31)	50.1 ± 35.0 (*n* = 27)	MD −12.21 (−30.64 to 6.22)	Welch t; 0.190
Mode of birth, *n* (%)	Vaginal: 22 (62.9%) Cesarean: 13 (37.1%)	Vaginal: 20 (64.5%) Cesarean: 11 (35.5%)	OR 1.07 (0.39 to 2.94)	Pearson χ^2^; 0.889
Infant sex, *n* (%)	Female: 18 (51.4%) Male: 17 (48.6%)	Female: 12 (38.7%) Male: 19 (61.3%)	OR 0.60 (0.22 to 1.59)	Pearson χ^2^; 0.300
Gestational age (weeks), mean ± SD	39.5 ± 2.4 (*n* = 35)	39.3 ± 2.5 (*n* = 31)	MD −0.20 (−1.41 to 1.01)	Welch t; 0.742
Parity, *n* (%)	Primiparous: 23 (65.7%) Multiparous: 12 (34.3%)	Primiparous: 16 (51.6%) Multiparous: 15 (48.4%)	OR 0.56 (0.21 to 1.50)	Pearson χ^2^; 0.245
GDM, *n* (%)	4 (11.4%)	7 (22.6%)	OR 2.23 (0.50 to 11.66)	Fisher exact; 0.324
PIH, *n* (%)	5 (14.3%)	3 (9.7%)	OR 0.65 (0.09 to 3.69)	Fisher exact; 0.713
Pre-pregnancy BMI (kg/m^2^), mean ± SD	23.4 ± 4.2 (*n* = 35)	24.7 ± 5.0 (*n* = 31)	MD 1.28 (−1.01 to 3.57)	Welch t; 0.269
BMI ≥ 25 kg/m^2^, *n* (%)	11 (31.4%)	11 (35.5%)	OR 1.20 (0.43 to 3.34)	Pearson χ^2^; 0.727
Hyperlactation, *n* (%)	27 (77.1%)	16 (51.6%)	OR 0.32 (0.11 to 0.91)	Pearson χ^2^; 0.030

Note: Values are mean ± SD or *n* (%). Mean differences (MDs) were calculated as HG − CG. Odds ratios (ORs) compare the odds in the HG with those in the CG for the first-listed category (vaginal birth, female sex, primiparity, or presence of the condition). Exact conditional 95% CIs are reported for GDM and PIH. Baseline comparisons were descriptive and were not included in the false discovery rate correction. BMI, body mass index; CI, confidence interval; CG, control group; GDM, gestational diabetes mellitus; HG, hypothyroidism group; PIH, pregnancy-induced hypertension.

**Table 2:** Between-group comparisons of human milk macronutrient composition and energy content.

Outcome	CG (*n* = 35), Mean ± SD	HG (*n* = 31), Mean ± SD	HG − CG Difference (95% CI)	Welch *t* (df)	*p*	q	Hedges’ g
Fat (g/100 mL)	3.57 ± 0.96	3.35 ± 1.09	−0.22 (−0.73 to 0.29)	−0.86 (60.10)	0.394	0.814	−0.211
Crude protein (g/100 mL)	1.41 ± 0.25	1.43 ± 0.27	0.02 (−0.11 to 0.15)	0.28 (62.33)	0.780	0.936	0.068
True protein (g/100 mL)	1.13 ± 0.19	1.15 ± 0.21	0.03 (−0.07 to 0.13)	0.52 (61.61)	0.606	0.909	0.127
Carbohydrates (g/100 mL)	7.81 ± 0.42	7.81 ± 0.40	−0.005 (−0.207 to 0.197)	−0.05 (63.71)	0.961	0.961	−0.012
Total solids (g/100 mL)	13.01 ± 1.03	12.76 ± 1.31	−0.24 (−0.83 to 0.34)	−0.84 (57.09)	0.407	0.814	−0.207
Energy (kcal/100 mL)	70.89 ± 8.64	67.42 ± 11.22	−3.47 (−8.45 to 1.52)	−1.39 (56.10)	0.169	0.814	−0.345

Note: Values are mean ± SD. Differences were calculated as HG − CG. *p* values were obtained using Welch’s two-sample *t* test. q values were calculated using the Benjamini–Hochberg false discovery rate procedure across the six outcomes. Energy was reported by the MIRIS analyzer using its macronutrient-based calculation algorithm. CI, confidence interval; CG, control group; HG, hypothyroidism group.

**Table 3:** Summary of exploratory analyses involving apparent milk TSH concentration.

Exploratory Analysis	Main Result	Interpretation
Study-group comparison of apparent milk TSH	CG: 0.0180 [0.0130–0.0208] µIU/mL (*n* = 35); HG: 0.0155 [0.0093–0.0210] µIU/mL (*n* = 31). Hodges–Lehmann location shift (HG − CG): −0.0020 µIU/mL (95% CI: −0.0070 to 0.0020); Mann–Whitney U = 459.0; *p* = 0.286; r = 0.131.	No statistically significant difference detected; values are exploratory apparent matrix measurements.
Hashimoto’s subgroup within HG	Hashimoto’s: 0.0100 [0.0091–0.0124] µIU/mL (*n* = 6); non-Hashimoto hypothyroidism: 0.0170 [0.0100–0.0210] µIU/mL (*n* = 25). Exact *p* = 0.208.	Hypothesis-generating only because the Hashimoto’s subgroup included six women.
Study group × pre-pregnancy BMI	Subgroups: CG, BMI < 25 (*n* = 24); CG, BMI ≥ 25 (*n* = 11); HG, BMI < 25 (*n* = 20); HG, BMI ≥ 25 (*n* = 11). Kruskal–Wallis H(3) = 2.73; *p* = 0.436.	Exploratory; overweight and obesity were combined because of sample size.
Study group × hyperlactation	Subgroups: CG, no HL (*n* = 8); CG, HL (*n* = 27); HG, no HL (*n* = 15); HG, HL (*n* = 16). Kruskal–Wallis H(3) = 4.69; *p* = 0.196.	Exploratory; hyperlactation was unevenly distributed and may reflect recruitment setting.
Overall hyperlactation comparison	HL: 0.0185 [0.0135–0.0210] µIU/mL; no HL: 0.0125 [0.0068–0.0220] µIU/mL. Mann–Whitney U = 637.0; *p* = 0.056.	Nominal trend only; not evidence of a biological effect.

Note: Values are medians [IQR] unless otherwise stated. CG, control group; HG, hypothyroidism group; HL, hyperlactation; IQR, interquartile range. Apparent milk TSH values should be interpreted cautiously because the assay was not formally validated in the human milk matrix.

## Data Availability

The data presented in this study are available on request from the corresponding author.

## Abbreviations

The following abbreviations are used in this manuscript:

ANOVA	analysis of variance
BMI	body mass index
CG	control group
HG	hypothyroidism group
ELFA	enzyme immunofluorescence assay
FT4	free thyroxine
GDM	gestational diabetes mellitus
HL	hyperlactation
PCOS	polycystic ovary syndrome
PIH	pregnancy-induced hypertension
Q–Q	quantile–quantile
SPSS	Statistical Package for the Social Sciences
T3	triiodothyronine
T4	thyroxine
TSH	thyroid-stimulating hormone
